# COVID-19 Antibody Levels among Various Vaccination Groups, One-Year Antibody Follow-Up in Two University Hospitals from Western and Central Turkey

**DOI:** 10.3390/vaccines12010059

**Published:** 2024-01-07

**Authors:** Mehmet Soylu, Pınar Sağıroğlu, Muhammed Alper Özarslan, Oğuzhan Acet, Zeynep Türe Yüce, Feyza İzci Çetinkaya, Seyfi Durmaz, Ömür Mustafa Parkan, Deniz Akyol, Ayşin Zeytinoğlu, Gamze Kalın Ünüvar, Meltem Taşbakan, Selma Gökahmetoğlu, Mustafa Altay Atalay, İsabel Raika Durusoy, Candan Çiçek, Hüsnü Pullukçu, Orhan Yıldız, Şaziye Rüçhan Sertöz, Memnune Selda Erensoy

**Affiliations:** 1 Department of Medical Microbiology, Faculty of Medicine, Ege University, Izmir 35100, Turkey; alperozarslan@hotmail.com (M.A.Ö.); candan.cicek@ege.edu.tr (C.Ç.); ruchan.sertoz@ege.edu.tr (Ş.R.S.); selda.erensoy@ege.edu.tr (M.S.E.); 2 Department of Medical Microbiology, Faculty of Medicine, Erciyes University, Kayseri 38039, Turkey; pinarsagiroglu@erciyes.edu.tr (P.S.); omurparkan@erciyes.edu.tr (Ö.M.P.); selmag@erciyes.edu.tr (S.G.); altay@erciyes.edu.tr (M.A.A.); 3 Department of Infectious Diseases, Faculty of Medicine, Ege University, Izmir 35100, Turkey; oguzhan.acet@ege.edu.tr (O.A.); denizakyol416@gmail.com (D.A.); gamzekalinunuvar@erciyes.edu.tr (G.K.Ü.); meltem.tasbakan@ege.edu.tr (M.T.); husnup@yahoo.com (H.P.); 4 Department of Infectious Diseases, Faculty of Medicine, Erciyes University, Kayseri 38039, Turkey; zeynepture@erciyes.edu.tr (Z.T.Y.); feyzaizci@erciyes.edu.tr (F.İ.Ç.); oyildiz@erciyes.edu.tr (O.Y.); 5 Department of Public Health, Faculty of Medicine, Ege University, Izmir 35100, Turkey; seyfidurmaz@gmail.com (S.D.); raika.durusoy@ege.edu.tr (İ.R.D.); 6 Department of Medical Microbiology, Faculty of Medicine, İzmir Economy University, Izmir 35330, Turkey; zeytinog@gmail.com

**Keywords:** SARS-CoV-2, coronavirus spike glycoprotein, mass immunizations, coronavirus nucleocapsid protein, healthcare workers, vaccine, BNT162, CoronaVac vaccine

## Abstract

Various clinical outcomes, reinfections, vaccination programs, and antibody responses resulted from the COVID-19 pandemic. This study investigated the time-dependent changes in SARS-CoV-2 antibody responses in infected and/or vaccinated and unvaccinated individuals and to provide insights into spike and nucleocapsid antibodies, which fluctuate during infectious and non-infectious states. This cohort study was carried out at the Ege University Faculty of Medicine hospital in İzmir (western Turkey) and the Erciyes University Faculty of Medicine hospital in Kayseri (central Turkey) between December 2021 and January 2023, which coincided with the second half of COVID-19 pandemic. The study included 100 COVID-19 PCR-positive patients and 190 healthcare workers (HCWs). Antibody levels were followed up via quantitative anti-SARS-CoV-2 spike and qualitative anti-nucleocapsid immunoassays (Elecsys™). Antibody levels declined after infection but persisted for at least 6–8 months. Individuals who had received only CoronaVac had higher anti-nucleocapsid antibody levels in the early months than those who received mixed vaccination. However, anti-spike antibodies persisted longer and at higher levels in individuals who had received mixed vaccinations. This suggests that combining two different vaccine platforms may provide a synergistic effect, resulting in more durable and broad-spectrum immunity against SARS-CoV-2. The study provides information about the vaccination and antibody status of healthcare workers in the second half of the pandemic and provides valuable insights into the dynamics of antibody responses to COVID-19 infection and vaccination.

## 1. Introduction

COVID-19 has shown variable clinical outcomes, leading researchers to investigate variations in antibody responses in different groups within the community following vaccination [1]. In addition, factors such as reinfection rates and the severity and duration of disease in reinfected cases have raised questions about the durability and efficacy of immune responses [2,3]. The use of different vaccine types has further complicated the study of antibody levels, as different vaccines have been found to induce different levels of antibodies against SARS-CoV-2 [4,5].

Maintaining adequate antibody levels is critical for long-term protection against COVID-19, and one of the most important concerns is determining the optimal frequency and number of vaccine doses required. In addition, understanding the longevity of these antibodies in peripheral blood is essential for the development of effective vaccination strategies. Early antibody responses against SARS-CoV-2, including immunoglobulin M (IgM), IgG, and IgA can be observed in sera approximately two weeks after symptom onset, with seroconversion typically occurring one week later. Antibodies to the spike (S) protein have been shown to have neutralizing properties and to persist longer than antibodies to the nucleocapsid (NC) protein [6,7]. On the other hand, antibodies targeting the nucleocapsid are produced early in the infection but decline rapidly during the disease [8].

Several manufacturers have developed antibody detection kits that can identify IgM, IgG and IgA antibodies to the SARS-CoV-2 spike and nucleocapsid proteins. These antibody-based assays measure the host’s humoral immune response to a recent or past infection and are detectable more than two weeks after the onset of symptoms. Optimal sensitivity and specificity of IgG and total antibody tests are typically achieved three to four weeks after symptom onset. Neutralizing antibody testing is essential, but its use requires specialized BSL-3 laboratories, which is a significant limitation. Recent research has shown promising correlations between virus specific immunoglobulin levels, particularly those targeting the S protein RBD, and viral neutralizing titers in convalescent plasma [9,10].

In Turkey, COVID-19 vaccination started in January 2021 with the CoronaVac vaccine (Sinovac Life Sciences Co., Ltd., Beijing, China), and approximately twelve months later the BNT162b2 vaccine (Pfizer/BioNTech, Mainz, Germany) was also introduced. With reinfections and different vaccination patterns, a heterogeneous population emerged. Recent studies claim that heterologous vaccination regimens elicit a strong immune response [11]. This study planned to bridge this gap, focusing particularly on the Turkish population, which has experienced unique challenges and vaccination strategies during the pandemic. In light of these considerations, the aim of this study was to investigate time-dependent changes in antibody responses in infected and/or vaccinated and unvaccinated individuals and to provide insights into spike and nucleocapsid antibodies, which fluctuate during infectious and non-infectious states.

## 2. Materials and Methods

This cohort study was carried out at the Ege University Faculty of Medicine hospital in İzmir (western Turkey) and the Erciyes University Faculty of Medicine hospital in Kayseri (central Turkey) between December 2021 and January 2023, which coincided with the second half of COVID-19 pandemic.

Study groups: two main groups, A and B, were included in this study.

Group A consisted of outpatients who tested positive for COVID-19 via PCR. Patients were followed up with SARS-CoV-2 S and NC antibody tests at day 0, 1 month, 4 months, and 6–8th month intervals after the date of positive PCR results.

Group B included two subgroups of health care workers (HCWs).

Group B1: HCWs who had a history of close contact, within one meter, with COVID-19-positive individuals, such as in the same workplace environment, living in the same house, kissing, handshaking, etc., and HCWs who were SARS-CoV-2 PCR negative 5–10 days after the exposure were included. Antibody levels were followed up with SARS-CoV-2 S and NC antibody tests at 0–4, 5–8, 9–12, and 13–17 months. Time intervals were evaluated depending on the latest vaccination dates of volunteers.

Group B2: Randomly selected asymptomatic HCWs without a known history of close contact with COVID-19 PCR-positive individuals in the last two weeks. This group was tested for SARS-CoV-2 S and NC antibodies using a single blood sample to represent the antibody status of HCWs in the study settings.

Antibody titer comparisons were evaluated depending on the latest vaccination dates of volunteers.

**Figure 1:**
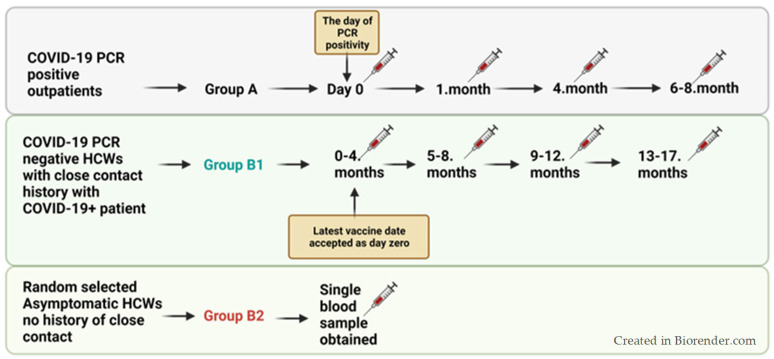
Study methodology showing included groups.

In order to investigate the relationship between antibody levels at the time of contact and the time elapsed since vaccination, the period from the date of the last vaccination of Group A and Group B1 until the time they were detected as COVID-19-positive (group A) or exposed to a COVID-19-positive person (group B1) was evaluated in three periods: 0–3 months, 4–6 months, and more than 6 months. Study methodology is shared in Figure 1.

The changes in anti-S and anti-NC antibody results were compared with the specified time periods, and statistical analysis using a *t*-test was performed to assess the significance of these changes. Also, individuals who experienced reinfection and/or were vaccinated during the study were excluded from the study.

### 2.1. Antibody Assays

The Elecsys^™^ Anti-SARS-CoV-2 S Quant Immunoassay (Roche Diagnostics, Rotkreuz, Switzerland) test was carried out using a Cobas e411 analyzer (Roche, Mannheim, Germany) and in accordance with the manufacturer’s recommendations. The test quantitatively measures the antibodies to the receptor-binding domain of the spike protein, with results reported in U/mL (10). Samples were classified as reactive (≥0.80 U/mL) or non-reactive (<0.80 U/mL) for SARS-CoV-2 RBD-specific antibodies. The dynamic range was 0.40–250 U/mL, with a lower detection limit of 0.35 U/mL. To analyze samples above 250 U/mL, Diluent Universal (Roche Diagnostics, Rotkreuz, Switzerland) was used to automatically dilute samples to 1:100. The analyzer multiplied the diluted data by the dilution factor, resulting in an upper quantitation limit of 25,000 U/mL for analysis. The U/mL values can be directly converted to binding antibody unit (BAU)/mL values, as specified by the first WHO International Standard for anti-SARS-CoV-2 immunoglobulin (NIBSC code: 20/136).

Elecsys^™^ Anti-SARS-CoV-2 (Roche Diagnostics, Rotkreuz, Switzerland) detects SARS-CoV-2 nucleocapsid antibodies (including IgG) in human serum and plasma. The test results were obtained using a Cobas e411 analyzer (Roche, Mannheim, Germany) and all samples were processed following the manufacturer’s recommendations. The test’s cut-off index (COI) values provided by the manufacturer indicate COI < 1 as negative and COI ≥ 1 as positive.

### 2.2. Statistical Analysis

SPSS version 25.0 (SPSS Inc., Chicago, IL, USA) was used for data analysis. Student-*t* and ANOVA tests were used to determine the correlations of antibody status and level with other variables. A paired-sample *t*-test was used to compare the antibody levels of the same participants between months. A ROC curve analysis was used to determine the antibody threshold level to predict a past infection. The significance level was taken as *p* < 0.05. ANOVA and paired sample *t*-test results were visualized and presented in graphs.

### 2.3. Ethics Statement

Ethical approval was obtained from the Medical Research Ethics Committee of the Ege University Faculty of Medicine on 8 December 2020 under number 20-12T/6.

## 3. Results

### 3.1. Features and Immunization Status of the Groups

The study group had a mean age of 39.05 years, with Group A having a mean age of 36.29, Group B1 having a mean age of 41.65, and Group B2 having a mean age of 39.5. Anagraphic features of the study group are shared in Table 1. A statistically significant difference among mean ages was evident between cohort groups (A and B1) (*p* = 0.001). In Group A, which consisted of 100 COVID-19 PCR-positive patients, 56 were female and 44 were male. When evaluated individually based on medical records, 80 cases were identified as having their first COVID-19 infection, while 20 cases had a history of at least one previous COVID-19 infection. Anti-S and anti-NC antibodies were evaluated for all COVID-19 PCR-positive cases. Due to a technical issue related to the diluent, the results of 17 cases where anti-S levels measured >250 U/mL on day 0 were not included in the antibody follow-up. Therefore, out of 100 COVID-19 PCR-positive cases, antibody levels were evaluated in 83 cases on day 0, in 77 volunteers in the 1st month, in 53 volunteers in the 4th month, and in 44 cases between the 6th and 8th months. Group B consisted of 132 females and 58 males, for a total of 190 HCWs. Group B1 included 88 HCWs who had a history of close contact with COVID-19-positive individuals, and Group B2 included 102 randomly selected asymptomatic HCWs without a known history of close contact with COVID-19 PCR-positive individuals in the last two weeks.

**Figure 2:**
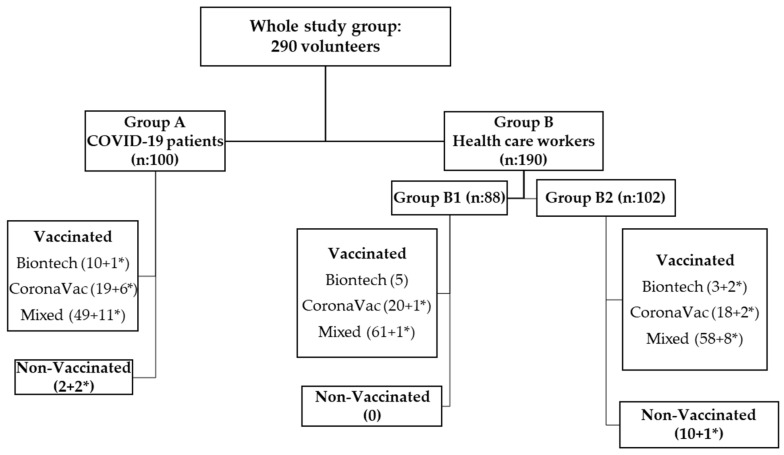
Whole study group distribution algorithm. Numbers in parentheses indicate the number of volunteers included in each study group. Numbers marked with an * indicate ones with a history of COVID-19 infection.

Vaccination data for Group A show that 49% of those who had their first COVID-19 infection received a mixed vaccination (BNT162b2 vaccine + CoronaVac), 19% received CoronaVac, 10% received the BNT162b2 vaccine, and 2% were non-vaccinated. Of those who had a previous COVID-19 infection, 11% had received a mixed vaccination, 6% had received CoronaVac, 1% had received the BNT162b2 vaccine, and 2% were non-vaccinated. The vaccination patterns and numbers of all cases are presented in Figure 2. Mean antibody levels and types of vaccination patterns for both Groups A and B are shown in Supplementary Table S2.

### 3.2. Relationship between Vaccination Patterns and Antibody Levels

**Figure 3:**
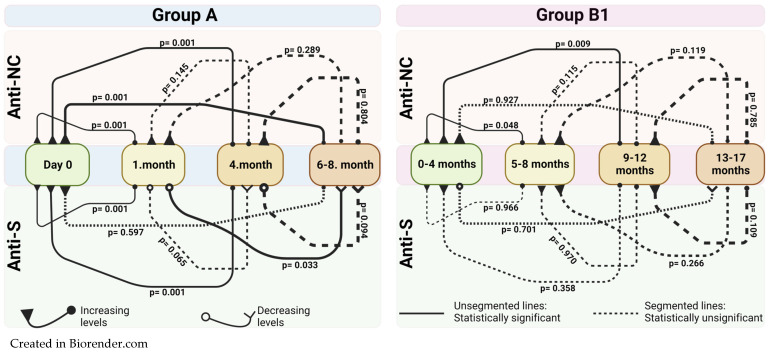
*p* values of mean difference in antibody levels between indicated months according to a paired sample *t*-test analysis of the cohort groups (Group A and Group B1). Unsegmented lines indicate statistically significant changes between antibody levels, segmented lines indicate no statistically significant change. Figure 2 is supported with Supplementary Table S1.

When Group A was evaluated, a significant increase in anti-S antibodies was observed between day 0 and 1 month (*p* < 0.001) and between day 0 and month 4 (*p* < 0.001), with a subsequent decrease between 1 and 6–8 months (*p* = 0.033). No significant difference was found in anti-S antibody levels between months 0 and 6–8 (*p* = 0.597). When comparing the anti-NC antibody levels of Group A, a significant increase was observed between day 0 and 1 month (*p* < 0.001), day 0 and 4 months (*p* < 0.001), and between day 0 and 6–8th months (*p* < 0.001). Further details on the significance of differences in other months are shown in Figure 3.

Comparing vaccinated individuals with and without a history of COVID-19 infection, those with a history had higher anti-NC antibody levels on day 0 (*p* < 0.001), while those without a history had significantly higher anti-S antibody levels at the 1st month (*p* = 0.011).

In Group A, individuals vaccinated only with CoronaVac had higher anti-NC antibody levels on day 0 compared to those with mixed-vaccination antibodies (*p* < 0.001). When comparing this group with individuals vaccinated only with the BNT162b2 vaccine, significant increases in anti-NC antibody levels were observed in mixed-vaccine recipients at the 1st and 4th months (*p* = 0.015 and *p* = 0.004, respectively). When comparing individuals vaccinated only with CoronaVac and those vaccinated only with the BNT162b2 vaccine, significantly higher Anti-NC ratios were observed in CoronaVac recipients at the 1st and 4th months (*p* < 0.001 and *p* = 0.027, respectively). In Group B1, anti-S levels were higher in individuals with mixed vaccinations compared to those vaccinated solely with CoronaVac at the 5–8, 9–12, and 13–17 month intervals; this data and the corresponding *p*-values are detailed in Table 2.

The antibody levels of the COVID-19 PCR-positive vaccinated group were compared with the results obtained from unvaccinated individuals. Higher levels of both anti-S and anti-NC antibodies were observed in the vaccinated group at all time periods except for day 0 anti-NC levels. *p* values are shared in Table 3.

Statistically significant differences were not obtained in anti-S antibody levels based on the time periods mentioned for Group B1. According to the *t*-test analysis for time-dependent changes in anti-NC levels in Group B1, statistically significant increases were obtained between 0–4th months and 5–8th months, as well as between 0–4th months and 9–12th months (*p* = 0.048 and *p* = 0.009, respectively). Mean differences in antibody levels between Groups A and B1 are shown in Figure 3.

When examining various vaccination patterns in the B1 group, individuals who received mixed vaccines had significantly higher levels of anti-S antibodies at 5–8 months, 9–12 months, and 13–17 months compared to those who received only CoronaVac (*p* = 0.003, *p* = 0.010, *p* = 0.010, respectively). Additionally, anti-NC antibody levels were significantly higher in the same group for the 0–4 and 5–8 month intervals among individuals vaccinated with CoronaVac (*p* < 0.001 and *p* = 0.017, respectively). No significant differences were found in the levels of anti-NC and anti-S antibodies between individuals who received either Pfizer/BNT162b2 or CoronaVac alone. However, higher levels of anti-S antibodies were evident in COVID-19-negative and asymptomatic vaccinated individuals compared to unvaccinated individuals across all time periods. There was no significant difference in anti-NC antibody levels. *p* values are shared in Table 3.

### 3.3. Effect of Time Elapse after Vaccination

**Figure 4:**
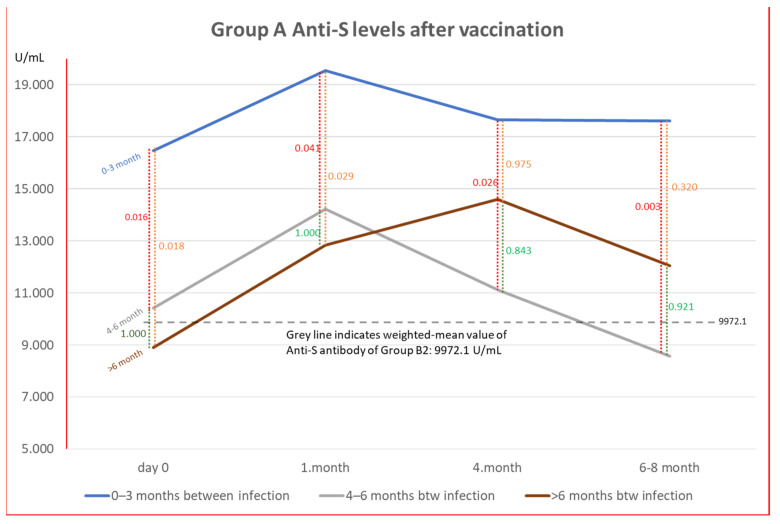
Trend of anti-S levels according to the time between the last vaccination and COVID-19 in Group A. Changes of antibody levels and *p* values on a monthly basis. Time intervals refer to last COVID-19 vaccination and time of infection. Segmented grey lines indicate the weighted mean antibody level of Group B2.

There was a significant difference (*p* < 0.05) in the mean anti-S antibody level of Group A between vaccine-to-disease intervals of 0–3 months and 4–6 months (mean difference (MD) = 6042.22 ± 2101.02), 0–3 months, and >6 months (MD = 7578.34 ± 2677.97). There was no significant anti-S antibody level difference between 4–6 months and >6 months between vaccination and disease (MD = 1536.12 ± 2587.70) (*p* > 0.05). In the first month after disease, there was a significant difference (*p* < 0.05) between 0–3 months and 4–6 months (MD = 5327.31 ± 2105.03) and 0–3 months and >6 months (MD = 6704.86 ± 2522.89). Four months after disease, there was a significant difference (*p* < 0.05) between 0–3 months and 4–6 months (MD = 6539.17 ± 2396.53). Six months after disease, there was a significant difference (*p* = 0.003) between 0–3 months and 4–6 months (MD = 9042.33 ± 2520.75). There was no statistically significant difference in anti-NC antibody levels in Group A and anti-S and anti-NC antibody levels in Group B1 for these three time periods. Depending on the last vaccination for Group A, time-dependent changes between antibody levels and *p*-values are shown in Figure 4. There were five cases in each of Groups B1 and B2 who received the BNT162b2 vaccine only. All the cases in Group B1 were anti-NC-positive, four were positive at the first visit, and only one was negative but later tested positive. In Group B2, three out of five cases were positive.

## 4. Discussion

At the outset of this study, we sought to address the question of the levels and duration of anti-S and anti-NC antibody positivity following breakthrough infections in different vaccination patterns, both in vaccinated and unvaccinated individuals. We were also able to see antibody levels in non-infected patients. Prior studies have indicated that antibodies generated against SARS-CoV and MERS-CoV viruses remain positive even a year after infection [12,13]. This prompted us to compare the expected levels of anti-SARS-CoV-2 antibodies and the anticipated decline in anti-S and anti-NC antibodies over time in various groups. Furthermore, the data indicating the short-lived nature of COVID-19 antibody responses reported in various studies, along with the variability in antibody levels across different vaccination and infection scenarios, the observation of antibody levels in COVID-19-positive cases of HCWs and a specific number of volunteers admitted to our hospital, and the discussion of these variables, constitute the backbone of this study [14].

Various studies have demonstrated that individuals who experience symptomatic COVID-19 tend to develop higher antibody levels compared to those who are asymptomatic [15]. Furthermore, a trend of decreasing antibody levels has been observed in infected individuals, typically starting around the third month after infection [16]. Notably, this decline is less pronounced in individuals who experienced asymptomatic infections [17]. In our study, ten volunteers had neither received a COVID-19 vaccine nor had a history of infection. However, we observed the presence of both anti-S and anti-NC antibodies in the sera of this group, which indicated that these volunteers might have had asymptomatic infections. When we compared the antibody levels of this group with those of the symptomatic COVID PCR-positive group, we found that PCR-positive patients had higher levels of antibodies, which is consistent with findings from various studies [18].

While the concept of immune exhaustion comes to the forefront in cases of severe symptoms, it raises questions about the relationship between the number of COVID-19 vaccine doses administered and antibody levels in individuals vaccinated with multiple vaccines over the long term. Additionally, the detrimental effects of severe COVID-19 infection on the development of lasting immunity might contribute to the inability to sustain long-term immune responses [19]. Various studies have indicated that a high IL-6 cytokine response can trigger inflammation and B lymphocyte exhaustion [20].

The kinetics of the development and maintenance of immune memory against SARS-CoV-2 indicate the emergence of specific memory B cells for 3–5 months, reaching a plateau stage every 6–8 months to support long-term antibody production among individuals who have survived COVID-19 [21]. The benefits and risks of excessive booster vaccination in COVID-19 survivors are still being debated. Some studies have found that it may not be necessary, while others have reported benefits, such as reduced risk of hospitalization and improved protection against variants [22]. Therefore, intended second booster vaccination dose might have a more adverse impact than an advantageous one in COVID-19 infected groups. This implies that an excessive boost in spike-specific immunity could potentially result in anergy and immune exhaustion [23]. A study by Goel et al. [24] emphasized the necessity of a single vaccine dose for individuals who had recovered from infection, while the second dose did not seem to increase the neutralizing antibody levels. Levi et al. [25] similarly emphasized the significance of administering a single vaccine dose to those who had recovered from infection to allow for the necessary immune response, and for this reason, a single vaccine dose was sufficient in symptomatic SARS-CoV-2-exposed subjects to reach a high titer of antibodies, suggesting no need for a second dose, particularly considering vaccine shortages. Immune response against emerging variants and the time after COVID-19 infection should be considered when determining new vaccination strategies.

Based on the findings of this study, CoronaVac vaccination led to an augmentation in both anti-S and anti-NC antibodies. Additionally, the time-dependent decline in antibody levels resulting from CoronaVac was not inferior to the mixed-vaccination approach. However, it is important to note that a comparison between CoronaVac and BNT162b2 responses is not appropriate due to the limited number of volunteers who received only the BNT162b2 vaccine. The presence of anti-NC-positivity in individuals vaccinated with BNT162b2 in HCW Group B1 who had no COVID-19 history might indicate asymptomatic infections or low viral loads. Takahashi et al. [26] indicated that stage of infection can lead to false-negative PCR results due to low viral load levels.

Evidence suggests that nucleocapsid antibody tests lose their reliability as indicators of previous infection beyond 6 months, post-infection. This phenomenon could stem from either the quicker decay kinetics of nucleocapsid antibodies or the increased background signal caused by cross-reactive nucleocapsid antibodies targeting other HCoVs [27]. However, the decline observed over the subsequent months also indicates that this might not provide prolonged protection. Anti-S antibodies on the other hand persisted longer and at higher levels in individuals who had received mixed vaccinations. This suggests a possible synergistic effect of combining two different vaccine platforms, providing a more durable and broad-spectrum immunity against SARS-CoV-2. The findings of this study suggest that combining inactive and mRNA COVID-19 vaccines could potentially emerge as the most optimal vaccination strategy. Cicek K et al. concluded that none of the models other than the homologous or heterologous vaccine models containing at least three doses of Pfizer-BioNTech vaccine were effective compared to those unvaccinated [28]. The current study gives information about the serological status and antibody fluctuations in healthcare workers and COVID-19 patients at two tertiary care hospitals in two different regions of Turkey, with the effects of homologous or heterologous vaccination doses and reinfections studied over time. According to previous data, including from the pre-vaccination era in Turkey, lower seroprevalence rates were reported among HCWs. However, seropositivity was significantly higher among HCWs compared to the general population [29]. While investigating the humoral immune response through serum IgG antibodies, we acknowledge the significant role of nasal IgA in mucosal defense against respiratory viruses like SARS-CoV-2 [30]. Resource constraints limited our current study, leaving a gap in understanding the complete immune picture at the viral entry point. Future research incorporating both IgG and IgA could provide a more comprehensive view of COVID-19 immunity, offering deeper insights into mucosal protection and potentially into reinfection dynamics.

In our study group, there were no associations between sex, age, and antibody levels. It should be noted that a significant portion of the participants in this study were healthcare workers; the absence of children and elderly individuals is one of the limitations of this study. Another limitation of this study is the underrepresentation of individuals vaccinated solely with the BNT162b2 vaccine. This imbalance could have influenced the comparisons made among different vaccination groups. Moreover, the study focused mainly on humoral immunity, leaving cellular immunity and cytokines, which are another critical component of the immune response against viruses, unexamined. Future studies should incorporate T-cell responses and memory B cells to provide a comprehensive view of immunity against SARS-CoV-2.

## 5. Conclusions

This study’s results highlight the heterogeneity in antibody responses during the pandemic, exacerbated by reinfections and varied vaccine regimens. Notably, we observed that S antibodies persisted longer than NC antibodies, although this observation warrants further verification. Our findings are particularly relevant in the context of vaccine types used. CoronaVac was predominant in our study group due to its early introduction to our population. Significantly higher S antibody levels were observed in individuals with mixed vaccinations (CoronaVac and BioNTech) compared to those vaccinated exclusively with CoronaVac. However, the limited number of participants vaccinated solely with BioNTech constrained our ability to conduct comprehensive comparisons. These insights underscore the need for ongoing surveillance of vaccine efficacy and antibody durability, particularly in the face of evolving viral strains and vaccination strategies.

## Figures and Tables

**Table 1:** Anagraphic features of the study group.

	Group A (n: 100)	Group B1 (n: 88)	Group B2 (n: 102)
Gender (F/M)	56/44	61/27	71/31
Age (mean value)	36.2	41.6	39.5
Vaccine subtypes			
1 dose BioNTech	0	2	0
1 dose CoronaVac	5	0	2
2 doses BioNTech	8	2	3
2d. CoronaVac	8	5	7
3d. BioNTech	3	1	2
3d. CoronaVac	10	13	8
4d. CoronaVac	2	3	3
1d. CoronaVac + 2d. BioNTech	0	1	0
2d. CoronaVac + 1d. BioNTech	19	15	17
2d. CoronaVac + 2d. BioNTech	35	44	39
2d. CoronaVac + 3d. BioNTech	4	2	9
3d. CoronaVac + 1d. BioNTech	2	0	1
Non-vaccinated	4	0	11

**Table 2:** *p* value comparison of mixed vaccination vs CoronaVac vaccinated groups.

Comparison of Group A: Mixed Vaccine vs. CoronaVac Only								
	Spike Antibodies				Nucleocapside Antibodies			
Time Intervals	Day 0	1st m.	4th m.	6–8th m.	Day 0	1st m.	4th m.	6–8th m.
***p* Values**	0.007	0.076	0.381	0.347	0.001	0.732	0.339	0.944
**Comparison of Group B1: Mixed Vaccine vs. CoronaVac Only**								
	**Spike Antibodies**				**Nucleocapside Antibodies**			
**Time Intervals**	**0–4th m.**	**5–8th m.**	**9–12th m.**	**13–17th m.**	**0–4 th m.**	**5–8 th m.**	**9–12 th m.**	**13–17 th m.**
***p* Values**	0.067	0.003	0.010	0.010	0.001	0.017	0.198	0.618

m.: month/months. *p* values were obtained by comparing the same time intervals.

**Table 3:** *p* value comparison of antibody levels obtained from COVID-19 PCR-positive and PCR-negative patients’ antibody test results between unvaccinated individuals.

Group A Compared to Non-Vaccinated Individuals								
	Spike Antibodies				Nucleocapside Antibodies			
Time Intervals	Day 0	1st m.	4th m.	6–8th m.	Day 0	1st m.	4th m.	6–8th m.
***p* Values**	0.001	0.001	0.001	0.001	0.687	0.003	0.001	0.001
**Group B1 Compared to Non-Vaccinated Individuals**								
	**Spike Antibodies**				**Nucleocapside Antibodies**			
**Time Intervals**	**0–4th m.**	**5–8th m.**	**9–12th m.**	**13–17th m.**	**0–4 th m.**	**5–8 th m.**	**9–12 th m.**	**13–17 th m.**
***p* Values**	0.001	0.001	0.001	0.002	0.785	0.452	0.356	0.148

m.: month/months. *p* values were obtained by comparing the same time intervals.

## Data Availability

The data presented in this study are available at reasonable request from the corresponding author.

## Supplementary Materials

The following supporting information can be downloaded at: https://www.mdpi.com/article/10.3390/vaccines12010059/s1, Table S1: Mean Antibody values of both groups (A and B); Table S2: has been added as Supplementary Material to provide a detailed analysis complementing Figure 3.

## Abbreviations

COVID-19	Coronavirus Disease 2019
S	Spike protein of SARS-CoV-2
NC	Nucleocapsid protein of SARS-CoV-2
RBD	Receptor binding domain
BAU	Binding antibody units
COI	Cut-off index
HCWs	Healthcare workers.
PCR	Polymerase chain reaction
SPSS	Statistical package for social sciences
MD	Mean difference
