# Supplementary material for: COVID-19 Antibody Levels among Various Vaccination Groups, One-Year Antibody Follow-Up in Two University Hospitals from Western and Central Turkey
Source: Vaccines (Basel). 2024 Jan 7;12(1):59. doi: 10.3390/vaccines12010059 (PMC10819475; doi:10.3390/vaccines12010059)
Supplement: Supplementary file 1 [file vaccines-12-00059-s001.zip › vaccines-2750659-supplementary.pdf]

Supplementary Table S1. Mean Antibody values of both groups (A and B). This table moved to supplementary section

MEAN ANTIBODY VALUES OF VARIOUS VACCINATION PATTERNS - GROUP A								
	**Day 0**		**1st month**		**4th months**		**6 to 8th months**	
	**Spike (U/mL)**	**NC (S/CO)**	**Spike**	**NC**	**Spike**	**NC**	**Spike**	**NC**
**1 dose CoronaVac**	1626.6 (5)	52.3 (5)	14514.5 (2)	77 (2)	11985 (1)	63.1 (1)	7573 (1)	230.5 (1)
**2 doses CoronaVac**	7345 (8)	20 (8)	15183.5 (6)	56.2 (6)	9095.4 (5)	59.4 (6)	6521.2 (4)	76.3 (4)
**3 d. CoronaVac**	10545.5 (9)	90.3 (9)	13482.6 (6)	129 (6)	8201.8 (5)	159.1 (5)	6328.4 (5)	134.8 (5)
**4 d. CoronaVac**	9218.1 (2)	36.9 (2)	12728 (2)	181.6 (2)	3755 (1)	27 (1)	12304 (2)	119.7 (2)
**2 d. Biontech**	7345 (8)	17.5 (8)	15183.5 (6)	56.2 (6)	9095.4 (5)	59.4 (5)	6521.5 (4)	76.3 (4)
**3 d. Biontech**	18853.6 (3)	0.7 (3)	23749 (3)	30.1 (3)	19680 (3)	57.06 (3)	23613 (3)	76.4 (3)
**2 d. CoronaVac + 1 d. Biontech**	10379.5 (19)	32.2 (19)	11757.2 (15)	142.8 (15)	13172.3 (6)	101 (6)	10497.7 (7)	92.6 (7)
**2 d. CoronaVac + 2 d. Biontech**	16457.6 (35)	25.7 (35)	18583 (26)	110.9 (26)	17604.8 (20)	149.8 (20)	16445.4 (16)	158.3 (16)
**2 d. CoronaVac + 3 d. Biontech**	8854.5 (4)	1.09 (4)	20889 (4)	198.8 (4)	15130 (4)	183.3 (4)	7101 (1)	190.1 (1)
**3 d. CoronaVac + 1 d. Biontech**	19630.5 (2)	149.3 (2)	18599 (2)	182.1 (2)	11514 (2)	203.8 (2)	7629 (1)	63.3 (1)
**Non-Vaccined**	5663.4 (4)	66.6 (4)	4626.7 (3)	62.2 (3)	None	None	5831 (1)	175.1 (1)

MEAN ANTIBODY VALUES OF VARIOUS VACCINATION PATTERNS - GROUP B1+ B2								
	**0-4 th months***		**5-8 th months***		**9-12 th months***		**13-17 th months***	
	**Spike (U/mL)**	**NC (S/CO)**	**Spike**	**NC**	**Spike**	**NC**	**Spike**	**NC**
**1 d. CoronaVac**	None	None	None	None	None	None	3425 (2)	126,2 (2)
**2 d. CoronaVac**	10365.4 (3)	98.2 (3)	13859.3 (3)	107.7 (3)	7334.8 (6)	160.8 (6)	8542.7 (6)	123.7 (6)
**3 d. CoronaVac**	11624.1 (13)	96.4 (13)	9329.4 (13)	86.8 (13)	10413.7 (10)	11.1 (6)	14259 (1)	58.1 (1)
**4 d. CoronaVac**	9402.2 (3)	197.1 (3)	7744 (6)	181.6 (6)	None	None	None	None
**1 d. Biontech**	None	None	14678 (2)	5 (2)	25000 (2)	92.2 (2)	16096 (1)	11.2 (1)
**2 d. Biontech**	4728.9 (2)	85.4 (2)	8944.7 (4)	117.7 (4)	5785 (2)	4.2 (2)	None	None
**3 d. Biontech**	16946 (3)	110.1 (2)	17120 (2)	103.6 (2)	None	None	None	None
**1 d. CoronaVac + 2 d. Biontech**	17929 (1)	0.77 (1)	16760 (1)	0.8 (1)	15846 (1)	40.3 (1)	None	None
**2 d. CoronaVac + 1 d. Biontech**	16097.1 (13)	44 (13)	9617.4 (23)	64 (23)	8607.8 (17)	121.6 (17)	13740.8 (5)	122.9 (5)
**2 d. CoronaVac + 2 d. Biontech**	15171.8 (49)	17.4 (49)	14723.7 (53)	71.5 (53)	13352.8 (35)	56.6 (35)	18726.5 (7)	41.6 (7)
**2 d. CoronaVac + 3 d. Biontech**	13259 (5)	84 (5)	14733.4 (7)	228 (7)	21823.6 (3)	43.8 (3)	None	None
**3 d. CoronaVac + 1 d. Biontech**			7110 (1)	146.8 (1)				

	**ANTI-SPIKE**	**ANTI-NC**						
**Non-Vaccinated and C**	2966.1 (11)	5.9 (11)						

(x) indicates the numbers of volunteers who donated blood sample for the study

d. :dose/doses

*: marked dates are indicating the time interval after the latest vaccine taken

Supplementary Table S2

	Day 0	1st month	4th months	6-8th months
**Day 0**		p<0,001 ↑	p<0,001 ↑	p= 0.597 ↑
**1st month**	p= 0,01 ↑		p= 0,065 ↑	p= 0.033 ↓
**4th month**	p= 0,01 ↑	p=0,345 ↑		p= 0,094 ↓
**6-8th months**	p= 0,01 ↑	p= 0,289 ↑	p= 0,804 ↑	

	0-4th months	5-8th months	9-12th months	13-17th months
**0-4th months**		p= 0,966 ↑	p= 0,358 ↓	p= 0, 701 ↓
**5-8th months**	p= 0,048 ↑		p= 0,970 ↓	p= 0,266 ↑
**9-12th months**	p= 0,009 ↑	p= 0,115 ↑		p= 0,109 ↑
**13-17th months**	p= 0,927 ↑	p= 0,119 ↑	p= 0,785 ↑	
